# Healthy Ground, Healthy Atmosphere: Recarbonizing the Earth’s Soils

**DOI:** 10.1289/ehp.124-A30

**Published:** 2016-02-01

**Authors:** Nancy Averett

**Affiliations:** Nancy Averett writes about science and the environment from Cincinnati, OH. Her work has been published in *Pacific Standard*, *Audubon*, *Discover*, *E/The Environmental Magazine*, and a variety of other publications.

On a bright October morning Dave Brandt tromps through the middle of his central Ohio wheat field. The grain was harvested months ago, but there isn’t an inch of bare dirt anywhere. Instead, more than 10 varieties of plants, including crimson clover, pearl millet, and Austrian winter peas, form a “cover crop cocktail” that stretches all the way to the road bordering his property. “This will be here all winter,” Brandt says. “And in the spring, we’ll plant corn right into this.”

Brandt hasn’t tilled his soil since 1972, when he rented his first 600 acres of farmland to grow wheat, corn, and soybeans. And by keeping plants on his land in various stages of growth and decomposition, Brandt appears to have increased the amount of carbon in his soil over the years. One study estimated that total organic carbon in the top foot of Brandt’s soil increased by 10% after six years of no-till, 35% after 20 years, and 61% after 35 years.^1^ (The data on which this estimate was based were not peer reviewed.) Overall, Brandt’s soil stored, or sequestered, an estimated average 960 kg of carbon per hectare per year.^1^

**Figure:**
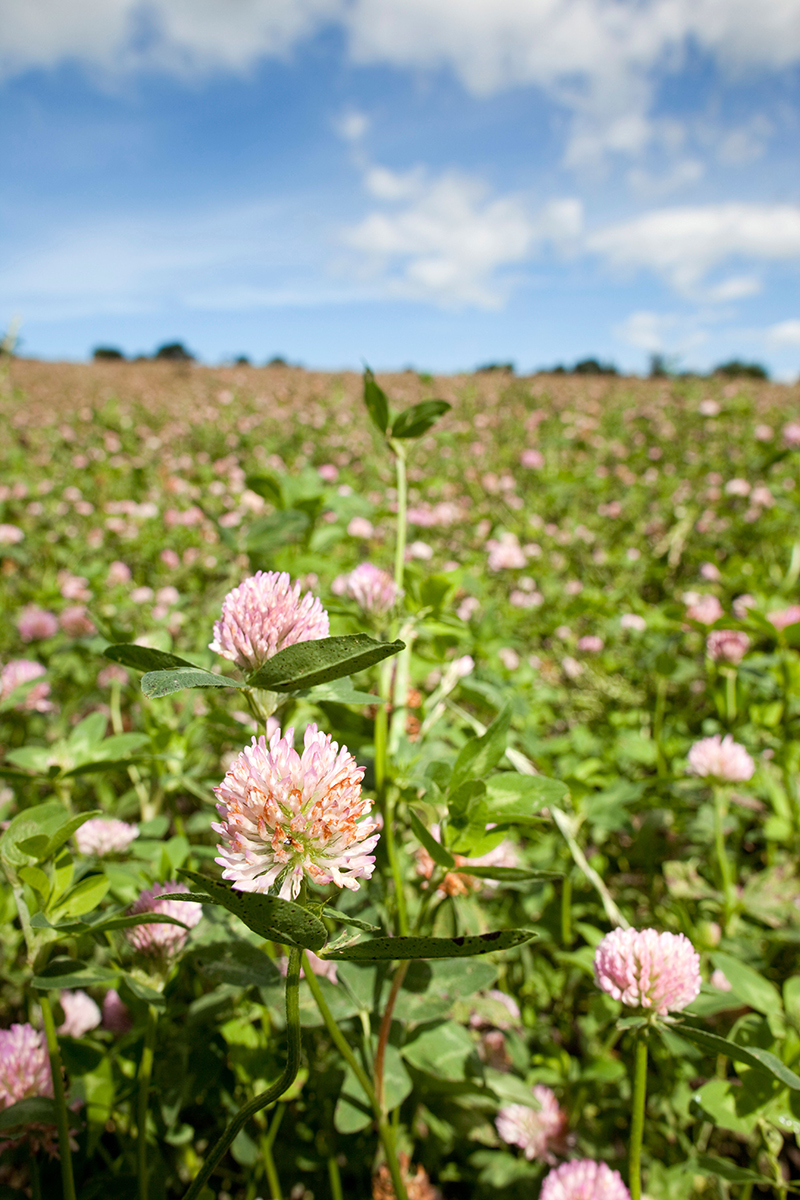
Since the dawn of agriculture, the world’s soils are estimated to have lost billions of metric tons of carbon to the atmosphere. Researchers are now looking at ways to recarbonize the global soil pool using methods such as cover cropping, no-till farming, and enriching the soil with biochar. Their goal: mitigating climate change. © David Chapman/Alamy Stock Photo

With figures like those, soil scientists and climate researchers believe that returning carbon to the soil on a large scale could help mitigate climate change. The world’s terrestrial carbon stores—the combined amount of carbon in soil and in plant matter—are much greater than the amount of carbon in the atmosphere: 3.12 trillion metric tons in the top meter of soil versus 780 billion metric tons in the atmosphere, by one estimate.^2^ Before the dawn of agriculture, there was even more carbon in the global soil pool—an estimated 55–78 billion additional metric tons,^3^ and the world’s plant biomass held still more, much of it lost to land use changes.^2^

That’s why Rattan Lal, one of world’s preeminent soil scientists and director of the Carbon Management and Sequestration Center (C-MASC) at The Ohio State University, has called for recarbonizing the world’s soils.^2^ Doing so, Lal says, “would be a truly win–win–win situation.” In addition to carbon sequestration, increasing carbon in soil has many other co-benefits: increased water storage in soil, increased length of the growing season, cooling of the ground via evapotranspiration, recharging groundwater aquifers, keeping springs and rivers flowing in the dry season. Soil itself filters water, reduces flooding, and provides a water reserve for plants in times of drought

**Figure:**
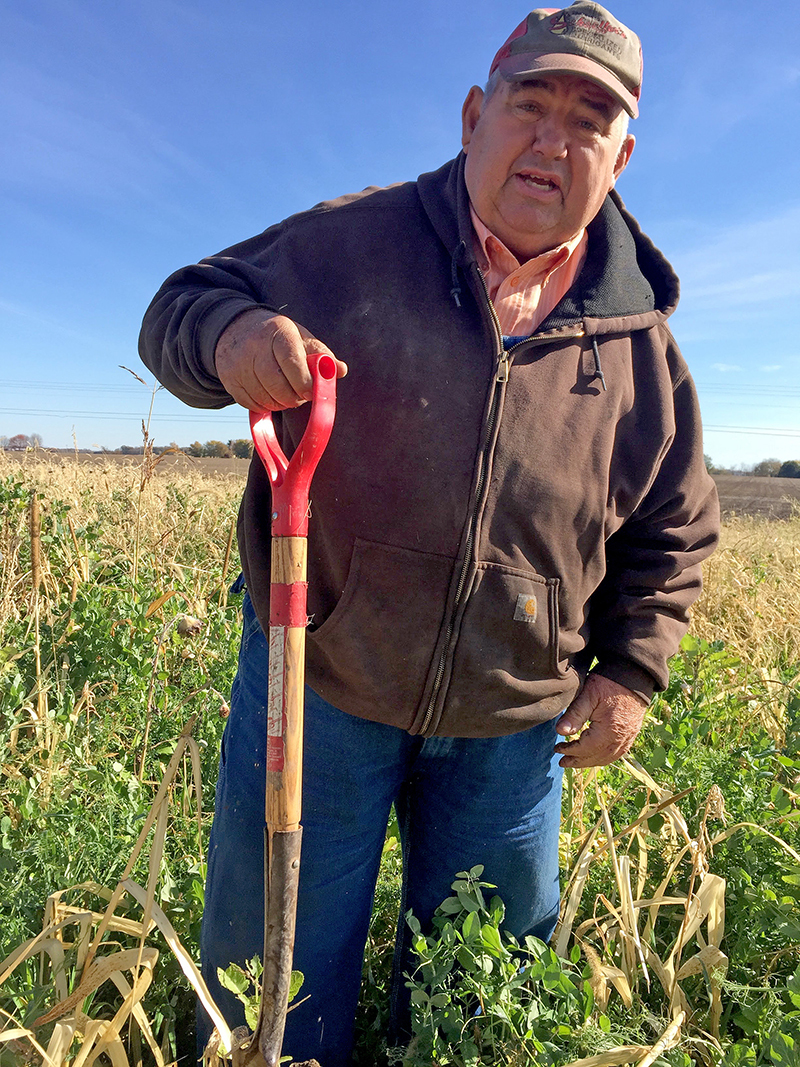
Dave Brandt has been practicing soil conservation measures on his Ohio farm since 1972. By one estimate, these measures sequestered an average 960 kg of carbon per hectare of his farmland per year. © Nancy Averett

Lal believes food security is an especially important co-benefit. By one estimate, about 24% of total global land area shows evidence of impaired productivity,^4^ and each year some 1–2.9 million hectares is degraded so badly that it becomes unsuitable for farming.^5^ Increasing population levels, predicted to reach 9.2 billion by 2050, will place more pressure on the world’s farmlands to produce enough food.^6^

Lal has calculated that increasing organic carbon in the soil surrounding plant roots by 1 ton per hectare per year can increase grain production by 32 million tons per year.^7^^,^^8^ “This is especially important for small landholders of sub-Saharan Africa, South Asia, and the Caribbean,” he says.

## Promising but Slow to Catch On

The same study that estimated the increased carbon levels in Brandt’s soils compared the productivity of two no-till fields on his farm—one with cover crops and one without—and found corn yields increased by 36–44% with the use of cover crops.^1^ In addition, Brandt estimates he uses 75% less fertilizer and herbicide on his land (he has eliminated pesticides altogether) and less fuel than if he were employing conventional methods that would require more passes over his fields. “We operate this farm on 2.5 gallons of diesel fuel an acre, compared to 35 gallons an acre for conventional farms,” he says.

Brandt also has observed no soil erosion, and he says his crops are protected against weather extremes. “Where we cover crop our soils,” he explains, “the soil never gets above 95–98°F in the summer, whereas in [his neighbor’s] conventionally tilled fields, the soil will get to 120–140°F.”

Despite those promising statistics, it has taken decades for Brandt to convince other local farmers to follow his lead, and many still resist. Indeed, just across the road lies a vista of bare brown soil on his neighbor’s farm; it will remain like that all winter. Nationally, no-till is used on about 13% of farms and cover crops on just 6%.^9^^,^^10^

Most conventional agricultural practices deplete rather than build up carbon.^11^ When farmers leave their fields bare between crops, for instance, only a small amount of organic matter is left to decompose and replenish the carbon stocks that are removed by harvesting the crops. The situation is worse in developing countries, where farmers often remove every bit of plant material left after harvest to feed animals or to burn as cooking fuel.^12^ In addition, tilling the soil brings any leftover plant material into contact with soil microbes faster than if the plant were to slowly degrade on the surface of the ground, which speeds up the plant’s decomposition and the return of its carbon stores to the atmosphere.^13^

But Brandt is feeling positive these days. That may be due to the fact that French Agriculture Minister Stéphane Le Foll made a special stop at his farm on a five-day swing through the United States in July 2015. Several months earlier, Le Foll had unveiled a new initiative that he would go on to present at the 21st Conference of the Parties to the United Nations Framework Convention on Climate Change (COP21), held in Paris in December. The initiative, dubbed 4/1000, calls for increasing the worldwide level of organic carbon in soil by a relative magnitude of 0.4% per year, an increase Le Foll says would compensate for current annual emissions of 4.3 billion tons of carbon dioxide (CO_2_) into the atmosphere.^14^^,^^15^

To discuss the potential benefits and challenges of soil carbon sequestration, Le Foll spent two days with Lal at Ohio State’s C-MASC, where the discussion focused on the feasibility of the 4/1000 proposal.^16^ C-MASC has collaborated with the Natural Resources Conservation Service (NRCS) and Agricultural Research Service of the U.S. Department of Agriculture to conduct research on this theme since early 1990s.

Lal and his colleagues then took Le Foll to Brandt’s farm so he could see a local operation that was successfully sequestering carbon. “He was here for about two hours,” Brandt says, clicking through some photos of the visit on his computer. “And he got real excited about what we’re doing.”

## Biochar: Another Opportunity to Increase Soil Carbon

Another option under exploration for increasing the carbon content of soil is biochar. This highly stable substance is produced when plant matter is heated at high temperatures in a low-oxygen environment, a process known as pyrolysis. Carbon is concentrated in the resulting biochar at levels twice that of ordinary plant materials.^17^

Biogeochemist Thomas J. Goreau, who is coordinator for the Soil Carbon Alliance information network, first encountered biochar years ago when someone brought him a sample of ash from rock layers marking the Cretaceous–Paleogene boundary, the geologic time period when scientists believe an asteroid hit the earth, causing massive fires and bringing 75% of plant and animal life to extinction. “When we looked at the ash through a microscope,” Goreau says, “you could see every cell in the plant that had burned. I mean, this sample was 65 million years old, and it was untouched.”

**Figure:**
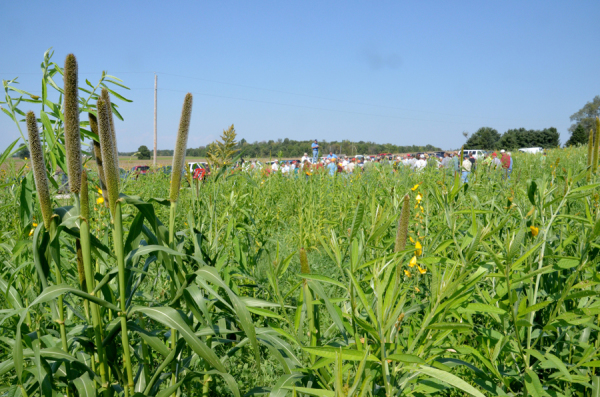
Brandt’s fields are covered with a “cover crop cocktail” of more than 10 plants species ranging in height from 4 inches to 4 feet. © Randall Reeder

**Figure:**
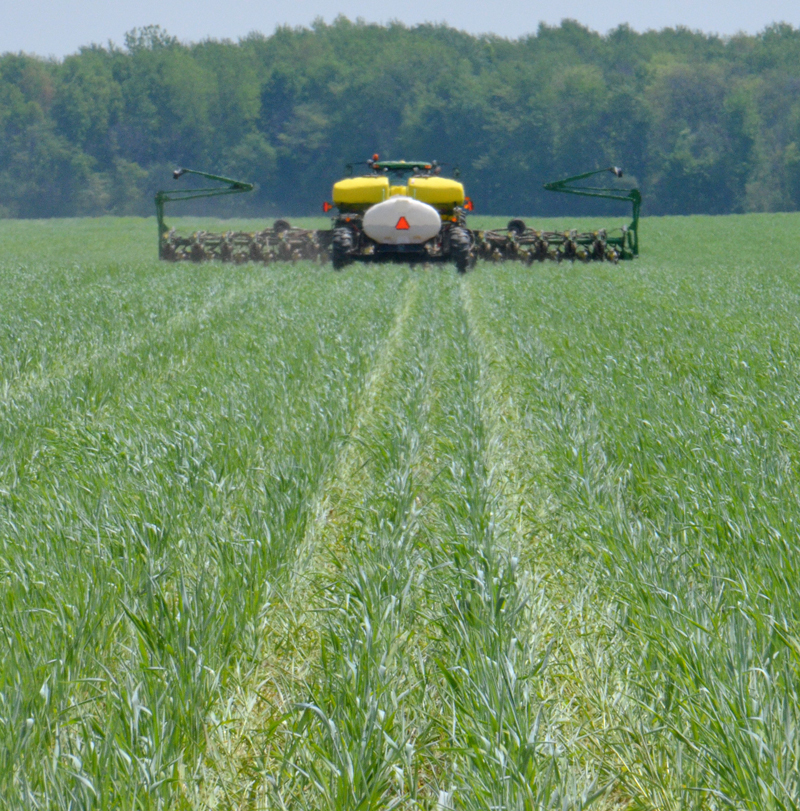
“Planting green” is the term used for planting cash crops into a standing live cover crop. In this photo corn is being planted directly into cereal rye on the Brandt farm. © Randall Reeder

**Figure:**
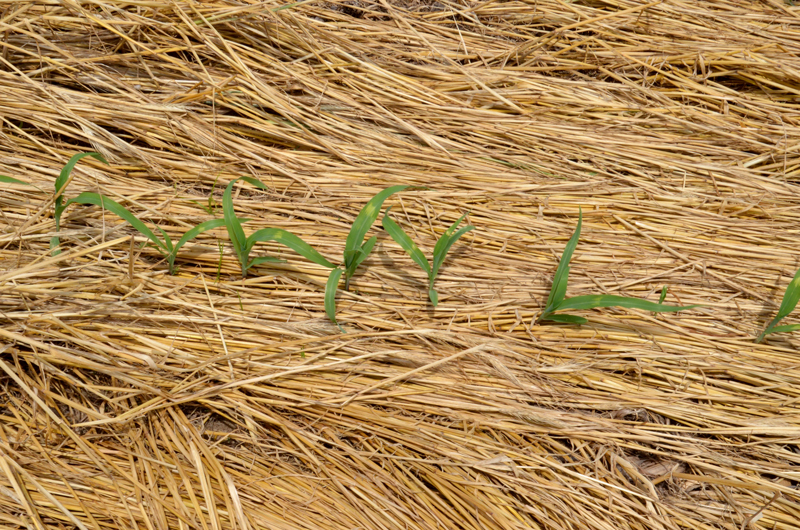
The cover crop then dies within a few days of planting the cash crop. Here corn is emerging through a mat of dead cereal rye. © Randall Reeder

He tells that story to illustrate just how long biochar can persist in the ground. Biochar deposits have helped produce extremely fertile soils, including the famous *terra preta* (“black earth”) soils in the Amazon.^18^ Yet, scientists say, it is only within the last decade—after the first international conference on the use of biochar to help mitigate climate change in 2007^19^—that researchers began to seriously study it.

**Figure:**
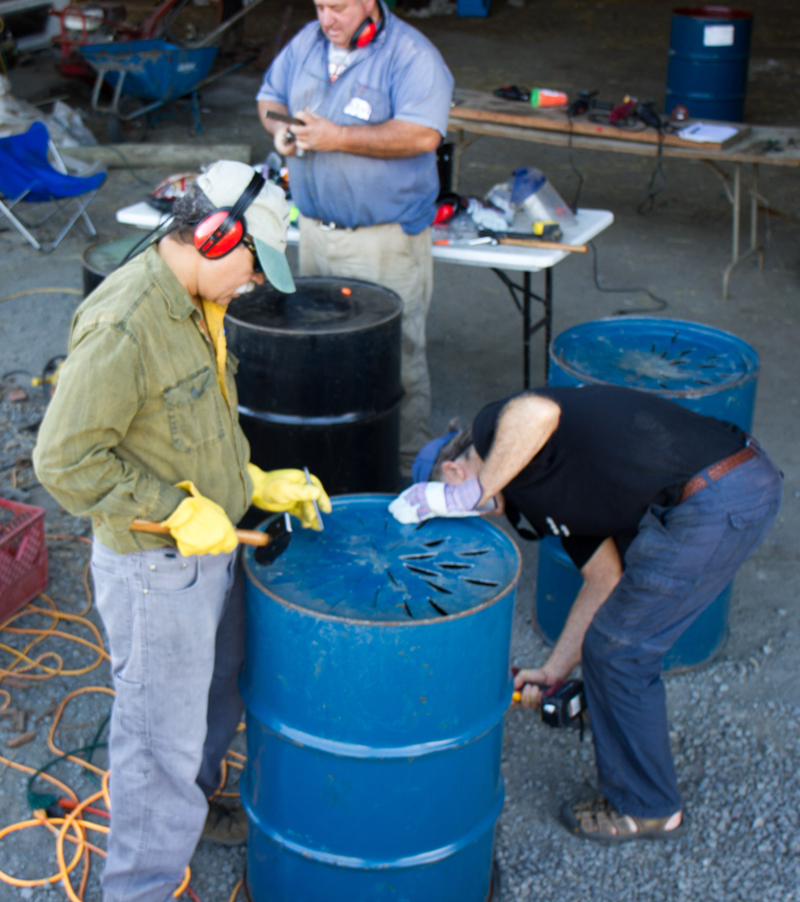
Participants at California’s Swallow Valley Farm Biochar School learn how to make their own biochar kilns. Biochar is produced when plant matter is heated at high temperatures in the absence of oxygen. © Josiah Hunt/Pacific Biochar

**Figure:**
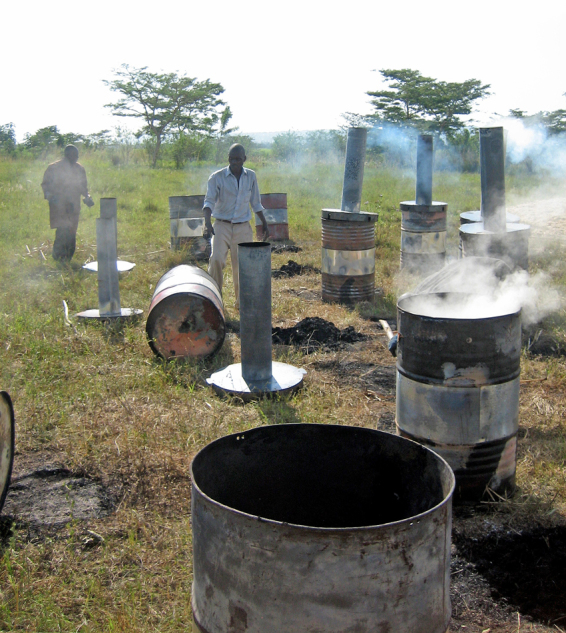
Biochar can be made in virtually any location from whatever plant matter is available; this Kenyan research project is using sugar cane waste. But biochar isn’t a one-size-fits-all solution. Farmers and land users must experiment with it to see if it works for their specific situation. © David T. Guerena/One Acre Fund

“If we look back, there were less than a dozen publications [on biochar] until 2007,” says Johannes Lehmann, a professor of soil science at Cornell University. Today, “more than 400 [are published] on the subject per year.”

Lehmann has coauthored a number of papers that show biochar can increase soil fertility and crop yields.^20^^,^^21^^,^^22^ He and colleagues have estimated that if farmers and land managers were to pyrolyze plant matter on their land (anything from crop residues to leaf litter) and bury the resulting biochar in their soils, they could potentially lock up 1 billion tons of carbon a year, which would offset the estimated 130 billion tons of CO_2_ that is expected to be added to the atmosphere over the next century.^23^ As an added benefit, renewable energy can be generated at the same time as biochar and partially displace fossil fuels, says David Laird, a professor in the Iowa State University Department of Agronomy—another step toward climate change mitigation.

Nevertheless, a range of socioeconomic, technological, and scientific challenges must be solved before there is widespread use of biochar. Right now, Lehmann says, the most important step is to have farmers and land users experiment with it and see how it works in their specific situation. Lehmann is working with farmers in Kenya, Sierra Leone, and Ethiopia to do just that. “We’re building cases for biochar, and I’m sure there will also be cases where biochar has not been found to add value,” he says. “Only when we tie all these cases together will we have a more realistic scenario of what could be done.”

## Reversing a History of Neglect

Convincing nations to undertake soil conservation measures of any type has been a long time coming. Lal and 30 other scientists co-authored a 2013 paper arguing that soil security has long been overlooked, pointing out that soil health was left off the agenda of numerous high-profile international meetings on climate change, sustainable development, ecosystem management, and biodiversity.^24^ Policies to protect soil are long overdue, says Andrea Koch, the lead author of the paper and director of the Soil Carbon Initiative at the University of Sydney’s U.S. Studies Centre. “Therefore,” she says, “soil degradation has continued silently to be a problem.”

Despite a long history of neglect, Koch says there are promising developments concerning soil security. In 2006 an international consortium of soil scientists undertook the massive project of compiling the world’s first digital soil map, known as GlobalSoilMap.^25^ The 3-dimensional map, slated for completion in 2018, will allow scientists to look at a range of soil properties—including pH levels, texture, and carbon levels—at a much finer scale than ever before. The data will also be available in a format that will make it easier for researchers in other fields, such as climatology, to incorporate accurate soil data into their research models.^26^^,^^27^

Having such information will be a boon to land managers and policy makers worldwide, says Thomas Reinsch, national leader of the World Soils Resources Branch of the U.S. Department of Agriculture’s NRCS. To make his point about the need for such information, Reinsch shares a story from a trip he and others took to Haiti to train agronomists how to recognize different soil properties. One day, Reinsch stood on a hilltop with Jean Pierre-Ogé, director of Forest and Soils for Haiti’s agriculture ministry. As the two looked out over the valley, Ogé pointed to an area where structures were being erected for families displaced by the 2010 earthquake. “Those soils over there are our best soils,” Ogé said, “[but] that’s where all of our houses are being built.”

Another boost came in 2011, when the Food and Agriculture Organization of the United Nations launched the Global Soil Partnership (GSP) to support sustainable management of soil resources for food security and climate change mitigation.^28^ Then, in 2012, the Institute for Advanced Sustainability Studies in Germany launched an annual Global Soils Week in Berlin, where researchers, farmers, and others meet to discuss soil issues.^29^

**Figure:**
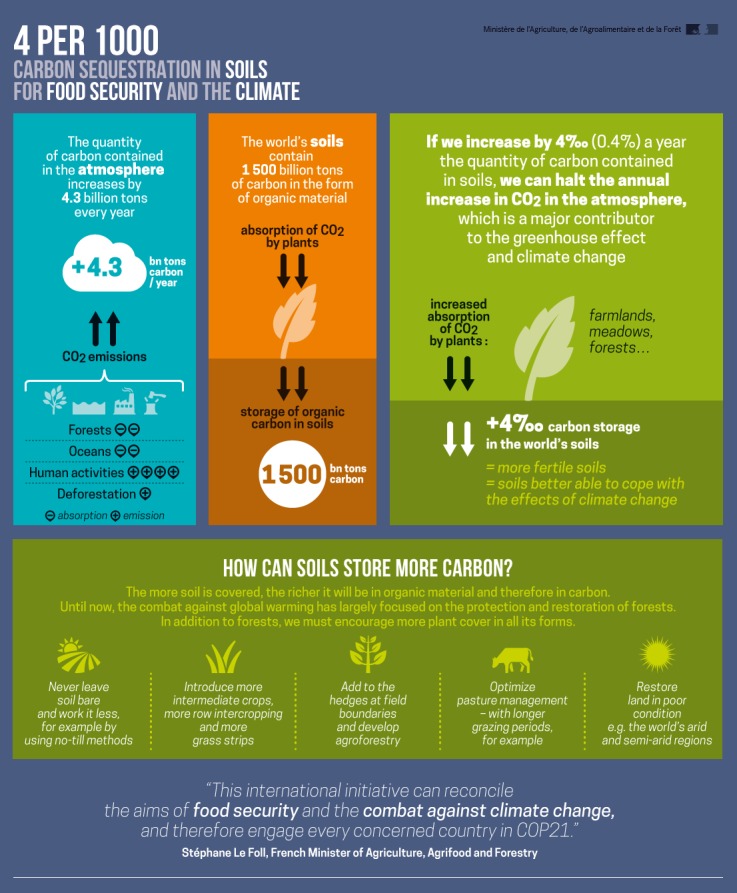
In December 2015 the 4/1000 initiative was adopted as a voluntary action plan under the Lima–Paris Action Agenda. © French Ministry of Agriculture

More recently, the NRCS launched a new Soil Health Division to promote a sea change in how farmers work their land by giving them technical advice, education, monetary incentives, and tools to adopt soil conservation measures such as no-till.^30^ NRCS scientists are working toward standardized soil health assessments to measure not just soil pH and nutrients but all its biological and physical properties such as water-holding capacity, compaction, microbial and enzymatic activity levels, porosity, and respiration rate.

Finally, the United Nations declared 2015 the International Year of Soil, a campaign to raise awareness, support policies, and promote investment toward soil security as well as enhance soil collection and monitoring.^31^ And on 4 December 2015, on the occasion of the 12th annual World Soil Day, the GSP released its first report on the state of the world’s soils.^32^

As for the 4/1000 initiative, many hoped that Le Foll would press for it to become part of the agreement signed by nations at the Paris climate talks in December. Instead, it was adopted as a voluntary action plan as part of the Lima–Paris Action Agenda,^33^ a set of events designed to keep momentum going on greenhouse gas reductions between meetings of the parties to the United Nations Framework Convention on Climate Change. Partners in 4/1000 agree to “implement farming practices that maintain or enhance soil carbon stock on as many agricultural soils as possible and to preserve carbon-rich soils.”^34^ A group of 25 nations and many other nongovernmental organizations, universities, and private companies have signed the agreement.^35^

## No Single Practice

For soil carbon sequestration advocates, this attention to soil health is long overdue. Goreau has long argued that the Intergovernmental Panel on Climate Change cannot seriously address climate change mitigation if it focuses only on reducing fossil fuels (the supply side of the equation, he says) and ignores increasing carbon sinks (the demand side).^36^ He calls the 4/1000 initiative a game changer. And he believes many countries will back it not because it’s the right thing to do, but because it’s in their own self interest. “And it is!” he says. “It’s hard to imagine that anyone’s going to lose from having their own land greener and more productive.”

Like many others in this area, Le Foll believes individual farmers are the most important ambassadors for carbon-enriching farming techniques such as no-till. After his July visit to C-MASC and Brandt’s farm, Le Foll invited Brandt to France so he could address a room full of international representatives at the Organisation for Economic Co-operation and Development. It was a heady experience for Brandt, and he likes to tell the story of an e-mail he received from one of Le Foll’s assistants asking him what he planned to wear to the meeting. “A dress shirt, a tie, and a new pair of black bibs,” he wrote back. “Those are my going-to-meetin’ clothes.” She replied, “Very funny, David. Buy a suit.”

Brandt roars with laughter at the memory. But after a moment, he turns serious. “Who would ever believe that what we’re doing”—he pauses, searching for the right words. He finally continues: “It’s quite humbling for me to think about a farmer talking to all those dignitaries about how to get [greater worldwide soil carbon sequestration] accomplished.”

Ultimately, there is no single practice—cover crops, no-till, biochar, or anything else—that is universally applicable to all the soils in the world. The overall strategy, Lal says, must be to create a positive soil carbon budget so that the input of carbon in soil exceeds the losses by erosion, decomposition, and leaching.

But that’s not enough, either. “Even if carbon sequestration is a win–win solution, in terms of climate change mitigation it remains a finite solution,” says agronomist Dominique Arrouays, scientific coordinator for the GlobalSoilMap project. “It should not prevent us from reducing fossil fuel emissions.”

**More than Carbon Depletion**

**Figure:**
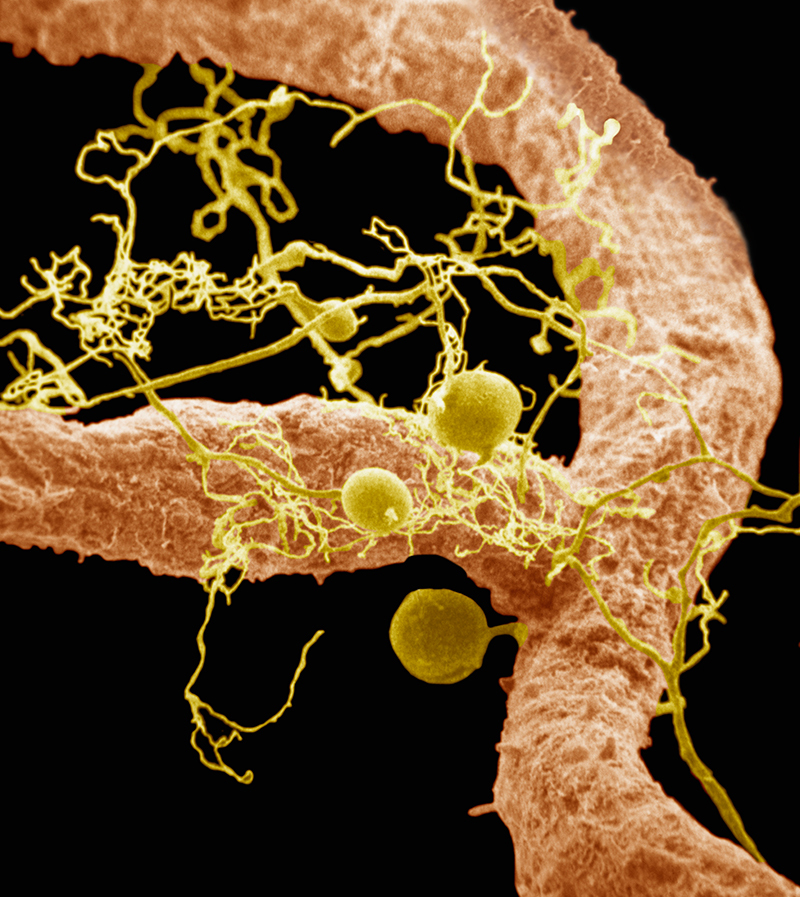
Fungal mycelia (yellow) help the roots of plants (orange) absorb nutrients from the soil. © Dr. Merton Brown/Visuals Unlimited, Inc.

Conventional agriculture practices don’t just deplete organic carbon levels; they can also reduce the amount of micronutrients in soil. Plants draw minerals toward the soil surface as they grow—on bare soil, these minerals are more readily lost through soil leaching and erosion.^37^^,^^38^ There is evidence that, as a result, plants growing in this soil—and the livestock that eat the plants—may contain lower amounts of the vitamins and trace minerals necessary for optimum human health.^37^^,^^39^

This is a potential problem, given that an estimated 2 billion people around the world are deficient in at least one of the 21 micronutrients that are essential for plant, human, and livestock health.^40^ Zinc deficiency—which kills an estimated 800,000 people a year and causes problems such as growth retardation and metabolic disorders in thousands of others—is the best documented.^37^ The most severe deficiencies happen in developing countries, where large segments of the population do not have access to a varied diet.^37^
